# A Phase 1 study of ARQ 087, an oral pan-FGFR inhibitor in patients with advanced solid tumours

**DOI:** 10.1038/bjc.2017.330

**Published:** 2017-10-03

**Authors:** K P Papadopoulos, B F El-Rayes, A W Tolcher, A Patnaik, D W Rasco, R D Harvey, P M LoRusso, J C Sachdev, G Abbadessa, R E Savage, T Hall, B Schwartz, Y Wang, J Kazakin, W L Shaib

**Affiliations:** 1 South Texas Accelerated Research Therapeutics, 4383 Medical Drive, San Antonio, TX 78229, USA; 2 Winship Cancer Institute of Emory University, 1365-C Clifton Road NE, Atlanta, GA 30322, USA; 3 Karmanos Cancer Institute, 4206—4th Floor HWCRC, 4100 John R, Detroit, MI 48201, USA; 4 Virginia G. Piper Cancer Center, Scottsdale Healthcare, 10460N 92nd Street, Scottsdale, AZ 85258, USA; 5 ArQule, Inc. One Wall Street, Burlington, MA 01803, USA

**Keywords:** FGFR inhibitor, solid tumours, FGFR2 fusion, phase 1

## Abstract

**Background:**

ARQ 087 is an orally administered pan-FGFR inhibitor with multi-kinase activity. This Phase 1 study evaluated safety, pharmacokinetics, and pharmacodynamics of ARQ 087 and defined the recommended Phase 2 dose (RP2D).

**Methods:**

Patients with advanced solid tumours received ARQ 087 administered initially at 25 mg every other day and dose-escalated from 25 to 425 mg daily (QD) continuous dosing. FGF19, 21, 23, and serum phosphate were assessed as potential biomarkers of target engagement.

**Results:**

80 patients were enrolled, 61 in dose-escalation/food-effect cohorts and 19 with pre-defined tumour types in the expansion cohort. The most common ARQ 087-related adverse events were fatigue (49%), nausea (46%), aspartate aminotransferase (AST) increase (30%), and diarrhoea (23%). Four patients (5%) experienced grade 1 treatment-related hyperphosphataemia. Dose-limiting toxicity was reversible grade 3 AST increase. The RP2D was 300 mg QD. Pharmacokinetics were linear and dose-proportional from 25 to 325 mg QD, and were unaffected by food. Statistically significant changes (*P*-value<0.05) suggest phosphate and FGF19 levels as markers of target engagement. In 18 evaluable patients with FGFR genetic alterations, 3 confirmed partial responses (two intrahepatic cholangiocarcinomas (iCCA) with FGFR2 fusions and one urothelial cancer with FGFR2 and FGF19 amplification) and two durable stable disease at ⩾16 weeks with tumour reduction (FGFR2 fusion-positive iCCA and adrenocortical carcinoma with FGFR1 amplification) were observed.

**Conclusions:**

ARQ 087 had manageable toxicity at the RP2D of 300 mg QD, showed pharmacodynamics effects, and achieved objective responses, notably in patients with FGFR2 genetic alterations.

Fibroblast growth factor receptors (FGFRs) have an important role in a variety of normal biological functions, including cellular proliferation, differentiation, migration, angiogenesis, and phosphate homeostasis (Cheng *et al*, 2011; Wesche *et al*, 2011). Dysregulation of the fibroblast growth factor (FGF)/FGFR tyrosine kinase family signalling axis has been implicated in a number of developmental syndromes and human malignancies, including gastric, breast, endometrial, bladder, small cell, and squamous non-small cell lung cancer, and intrahepatic cholangiocarcinoma (iCCA; Wu *et al*, 2013; Helsten *et al*, 2015, 2016). In human cancers, FGFRs can be deregulated by multiple mechanisms, including aberrant expression, amplifications, mutations, translocations, and fusions (Wu *et al*, 2013; Katoh, 2016). These pathogenic alterations have fueled significant interest in the FGFR pathway as a target for therapeutic intervention, with several drugs in development but none yet specifically approved for FGFR-driven tumours (Andre *et al*, 2013a, 2013b; Dienstmann *et al*, 2014; Soria *et al*, 2014; Tabernero *et al*, 2015; Nogova *et al*, 2017).

ARQ 087 is an orally administered adenosine triphosphate (ATP)-competitive pan-FGFR inhibitor with multi-kinase activity. In biochemical studies, ARQ 087 showed potent activity against both wild-type and variants of the *FGFR* kinases (*FGFR1-3*), and to a lesser extent against *FGFR4*, with inhibitor concentration required for 50% inhibition (IC_50_) values in the low nM range for FGFR family members. Activity within a five-fold range of the IC_50_ of FGFR2 was observed in KIT, PDGFR, RET, and SRC family members, but ARQ 087 had limited activity against other kinases tested (Hall *et al*, 2016). In Ba/F3 cell proliferation assays, except for FGFR1–2, FGFR fusions, KIT, LCK, and ARG, the majority of kinases had growth inhibition of 50% values above 1000 nM (Hall *et al*, 2016). Further, in an autophosphorylation assay, ARQ 087 inhibited autophosphorylation of FGFR1 and FGFR2 in a dose-dependent manner, suggesting that, in addition to inhibiting the active (phosphorylated) form of the kinase, ARQ 087 binds to the unphosphorylated or inactive form of the kinase and delays its activation. Preclinical studies demonstrated potent inhibition of tumour growth in FGFR pathway-activated models, including in FGFR2-driven (amplification/fusion/mutation) tumour xenografts (Hall *et al*, 2016).

The primary objective of this phase 1 study (NCT01752920) was to assess the safety and tolerability of ARQ 087 in patients with advanced solid tumours. The secondary objectives included determination of the safe and biologically active recommended phase 2 dose (RP2D), pharmacokinetics (PK), pharmacodynamic (PD) effects, and preliminary efficacy of ARQ 087.

## Materials and methods

This phase 1 study was conducted in accordance with all applicable local regulatory requirements and laws. All enrolled patients signed an institutional review board-approved informed consent form.

### Patients

Patients with histologically or cytologically confirmed advanced, inoperable, or metastatic solid tumours who failed to respond to standard therapy or for whom standard curative therapy does not exist; who were ⩾18 years of age; had radiologically evaluable or measurable disease; had Eastern Cooperative Oncology Group performance status ⩽2; and had adequate bone marrow, liver, renal, and cardiovascular function were eligible. Patients who underwent chemotherapy, radiotherapy within 28 days of study commencement, and previous anticancer therapy with FGFR inhibitors, or those who had unstable CNS metastases and a history of or current clinically significant disorders (e.g., myocardial infarction less than 6 months before enrolment and active HIV infection) were excluded.

### Study design

This is a first-in-human, open-label, multicenter phase 1/2 dose escalation, food-effect, and expansion/signal finding study. The ARQ 087 starting dose of 25 mg QOD, administered orally in a fasting condition, was determined based on non-clinical toxicology data. Treatment cycles were continuous 28-day periods without treatment interruption.

In phase 1 of the study, patients with unselected solid tumours were enrolled in the dose-escalation and food-effect cohorts. Once a potential RP2D was defined, only patients with cholangiocarcinoma and adrenocortical carcinoma independent of the molecular characteristics, and other solid tumours harbouring *FGFR1-3* genetic alterations or *KIT/PDGFR* mutations were eligible for enrolment. Dose-escalation was performed according to the standard 3+3 dose-finding schema and continued until the maximum tolerated dose (MTD), the highest dose level at which fewer than 33% of enrolled patients experienced dose-limiting toxicity (DLT) in the first treatment cycle, was reached. Up to three additional patients eligible for paired biopsies could be enrolled at dose levels declared safe. Patients not completing the first cycle for reasons other than DLT were considered inevaluable and replaced. The protocol was subsequently amended to include only selected patients with tumours having confirmed FGFR mutations or translocations, including iCCA with FGFR2 gene fusion; the Phase 2 part of the study is ongoing and will be reported separately.

DLT was defined as a grade ⩾3 haematologic or non-haematologic ARQ 087-related toxicity observed during cycle1: haematologic toxicities included grade 4 anaemia, thrombocytopaenia, neutropaenia; grade⩾3 neutropaenia with fever (⩾38 °C/100.4 F) or lasting longer than 7 days despite optimal treatment, grade⩾3 thrombocytopaenia in the presence of bleeding; and any grade ⩾3 non-haematologic toxicities except nausea, vomiting, or diarrhoea responding to optimal medical management within 48 h. Patients experiencing a DLT were discontinued from study or continued with dose reduction as deemed appropriate. Patients received ARQ 087 until disease progression, unacceptable toxicity, investigator decision, or consent withdrawal.

### Study assessments

Safety assessments were performed at baseline and weekly during the first cycle and every 2 weeks thereafter throughout the study, and included physical examinations, vital sign measurements, clinical laboratory tests, 12-lead electrocardiogram (ECG), urinalysis, and collection of AE information. Patients previously treated with anthracyclines had left ventricular ejection fraction measurement performed every 8 weeks. Treatment-emergent AEs (TEAEs) were graded using Common Terminology Criteria for Adverse Events (CTCAE) version 4.03. The grading of hyperphosphataemia, not included in the CTCAE, was Study defined, with confirmed hyperphosphataemia >ULN-7 mg dl^−1^ assessed as grade 1, >7 mg dl^−1^ as grade 2, and >9 mg dl^−1^ as grade 3. Management of hyperphosphataemia was according to institutional guidelines/Investigators’ discretion.

Radiologic assessments of tumour response by computed tomography or magnetic resonance imaging were conducted at baseline and approximately every 8 weeks thereafter according to RECIST version 1.1 (Eisenhauer *et al*, 2009).

### Pharmacokinetic study

Serial blood samples were collected for evaluation of PK parameters on days 1 and 22 (pre-dose and 1, 2, 4, 6, 8, 10, 12, and 24 h after ARQ 087 dosing), day 8 (pre-dose), and day 15 (pre-dose) of cycle 1; on day 1 and day 15 of all subsequent cycles (pre-dose); and at end of treatment. Pharmacokinetic parameters were computed using non-compartmental methods with Phoenix version 6.4.0.768, (Certara USA Inc., Princeton, NJ, USA). Blood samples were centrifuged, and the plasma was separated and stored at −20 or −80 °C until analysis was performed. All sample analyses were performed by Covance Inc. (Indianapolis, IN, USA) using a validated HPLC-MS/MS method (ArQule, Burlington, MA, USA, on file).

### PD assessments

Blood samples were collected for evaluation of soluble PD markers such as serum phosphate and plasma FGF19, 21, and 23. FGF samples were collected pre-dose on days 1, 8, 15, 22 of cycle 1 and on day 1 of cycles 2–5. FGF19, 21, and 23 were measured at ArQule using commercially available ELISA kits (EMD Millipore, Billerica, MA, USA; R&D systems, Minneapolis, MN, USA). All kits were validated for internal use at ArQule, and data used for assessment of potential target engagement. PD parameters including maximum observed response value (*R*_max_), maximum per cent change from baseline (*B*) response value (%B*R*_max_) calculated as [*R*_max_−*B*]/*B ×* 100, area of the response curve that is above the baseline effect value from time point zero (pre-dose) up to C1D22 (AUEC Above_0–t_), area of the response curve that is below the baseline effect value from time point zero (pre-dose) up to C1D22 (AUEC Below_0-t_), and net area of the response curve above and below the baseline effect value baseline calculated as AUEC Above_0–t_−AUEC Below_0–t_ (AUEC Net_0–t_) were computed using Phoenix version 6.4.0.768, (Certara USA Inc.). Tumour samples were evaluated for FGFR genetic alterations by mutational analysis, array comparative genomic hybridisation, or next-generation sequencing at local or central laboratories using standard protocols. Paired tumour biopsies (baseline and cycle 2 day 1±7; optional for patients enrolled in Dose Escalation cohorts and mandatory for patients enrolled in the Expanded cohort) were collected to evaluate changes in pFGFR, pFRS2*α*, and pERK status by standard immunohistochemistry (ArQule, in-house data).

### Statistical analysis

Descriptive statistics was used for the analysis of demographic, PK, safety, and the anti-tumour activity data. Patients receiving at least one daily dose of ARQ 087 were considered evaluable for safety analyses. Patients who have received at least one cycle of ARQ 087 and had at least one disease assessment following the initiation of therapy were considered evaluable for response.

## Results

### Patients

A total of 80 patients were enrolled at four sites in the United States between December 2012 and October 2015 and received at least one dose of ARQ 087 – 61 patients in the dose-escalation/food-effect cohorts and 19 patients in the expansion cohort. Patient demographics and baseline characteristics are shown in Table 1. FGFR genetic alterations were documented in 22 patients. The dose-escalation phase initially tested ARQ 087 in patients at 25 mg QOD (*n*=3), and then at 25 mg QD (*n*=6), 50 mg QD (*n*=6), 100 mg QD (*n*=4), 150 mg QD (*n*=5), 200 mg QD (*n*=5), 250 mg QD (*n*=7), 325 mg QD (*n*=6), and 425 mg QD (*n*=7). Additional patients were dosed at 400 mg QD under fasted (*n*=5) or fed (*n*=7) conditions. Following review of safety and PK data, 19 patients were enrolled in the expansion cohort and were treated with ARQ 087 at 300 mg QD under fasted condition.

At the time of the data cutoff, 78 patients (97%) had discontinued study treatment, including 47 patients with radiologic disease progression, 17 patients with clinical progression, and 8 patients due to adverse events, including 2 patients with DLT, 4 patients due to lack of clinical benefit, and one patient each due to decision of the investigator or withdrawal of consent. The median duration of drug exposure for all patients was two cycles (range: 0–43 cycles).

### DLT and MTD

DLT of increased aspartate aminotransferase (AST, grade 3) occurred in one patient at 250 mg QD and two patients in the 425 mg QD cohort. AST levels returned to baseline following drug interruption and dose modification (dose was reduced from 250 to 200 mg; no recurrence was observed after rechallenge) or discontinuation. The MTD was determined as 400 mg QD, with no DLTs at this dose in 12 patients, whether under fed or fasted conditions.

### Safety

TEAEs were reported in 79 patients (99% Table 2). The most common TEAEs were fatigue (58%), nausea (54%), AST increase (36%), decreased appetite and diarrhoea (29%, each), vomiting (28%), and constipation (25%). The most common AEs that were considered ARQ 087-related were fatigue (49%), nausea (46%), AST increase (30%), and diarrhoea (23% Supplementary Table 1).

TEAEs ⩾grade 3 were observed in 39 out of 80 (49%) patients; 14 (18%) were considered treatment-related, including two cardiac events of abnormal ECGs, respectively, with QTc prolongation and non-ischaemic diffuse T-wave inversion, that were also reported as serious adverse events (SAE; Table 2). There were eight TEAEs in eight patients (10%) that led to treatment discontinuation, of which three (two events of grade 3 AST increase and one event of grade 3 abnormal ECG) were considered to be related to ARQ 087. Of 28 SAEs only two, as noted above, were reported as ARQ 087-related. Six deaths (five due to disease progression and one due to cardiomyopathy) occurred within 30 days of the last dose administration, and none were considered related to ARQ 087.

Hyperphosphataemia (grade 1) was reported in four patients (one patient each 200 and 425 mg QD; two patients 300 mg QD). With the exception of two patients treated with hydration and phosphate-binding therapy for hyperphosphataemia, no other patients required specific intervention or dose interruption. Although serum phosphate levels increased in the majority of patients, they typically plateaued during the first cycle and remained elevated for the duration of treatment. Grade 1 nail toxicity, including nail discolouration and onychomycosis, was reported in five patients (6%). In three patients, three events of eye toxicity were reported as ARQ 087-related: dry eye (grade 1, *n*=1), blurred vision (grade 1, *n*=1), and visual impairment (grade 2, *n*=1). Ophthalmologic examination of the patient with blurred vision revealed cataracts and vitreous degeneration; blurring resolved with corrective lenses. The patient with visual impairment of the left eye had symptoms atypical for central serous retinopathy (CSR), and had rapid resolution following brief dose interruption. She did not agree to ophthalmic evaluation; thus, CSR could not be definitively excluded.

On the basis of cumulative safety data showing a dose-dependent increase in overall number of drug-related AEs and lack of any significant difference in PK exposure when dose levels were increased from 250 to 425 mg QD, the dose of 300 mg QD was defined to be the RP2D.

### PK results

**Figure 1:**
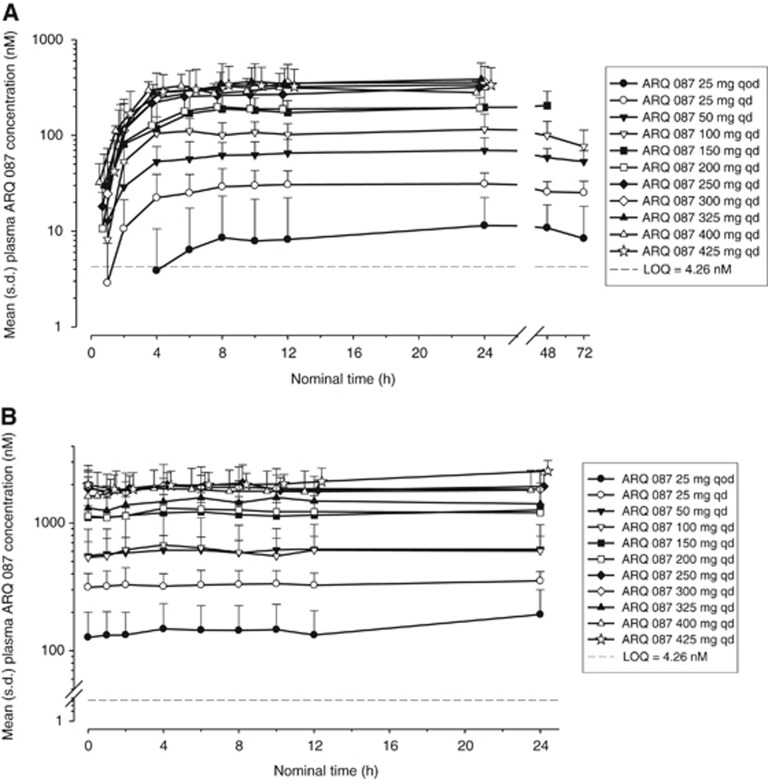
**Pharmacokinetics of ARQ 087.** Mean (+s.d.) plasma concentrations of ARQ 087 vs. time after (**A**) a single oral dose (day 1) and (**B**) multiple doses (day 22) of ARQ 087 (semi-log scales).

Following a single oral dose of ARQ 087 at dose levels ranging from 25 mg QOD to 425 mg QD, ARQ 087 was absorbed slowly, with the mean peak plasma ARQ 087 concentrations (*T*_max_) generally observed at 8-h post-dose. ARQ 087 plasma concentration declined in a mono-exponential manner over 24- to 72-h post-dose. The mean concentration–time profiles of ARQ 087 showed higher concentrations on Day 22 compared with those observed after the first dose at comparable dose levels, suggesting approximately five times accumulation of ARQ 087 following repeated dosing (Figure 1).

PK parameters are presented in Table 3 (Supplementary Table 2 for full data set). After oral daily doses of ARQ 087 for a 22-day period, the mean rate and extent of ARQ 087 exposure increased when the dose of ARQ 087 increased from 25 mg QD to 250 mg QD. However, exposure between 250 mg QD and 425 mg QD was similar, suggesting that absorption was saturated at dose levels above 250 mg QD. On day 22, the mean peak plasma drug concentration (*C*_max_) ranged from 330 to 913 ng ml^−1^ and the mean area under the plasma concentration–time curve to the last measurable concentration (AUC_last_) ranged from 6690 to 19 887 ng.h ml^−1^ for the 100 and 400 mg dose levels. In addition, in the range of 100–300 QD the half-life of ARQ 087 was ∼5 days.

### PD results

**Figure 2:**
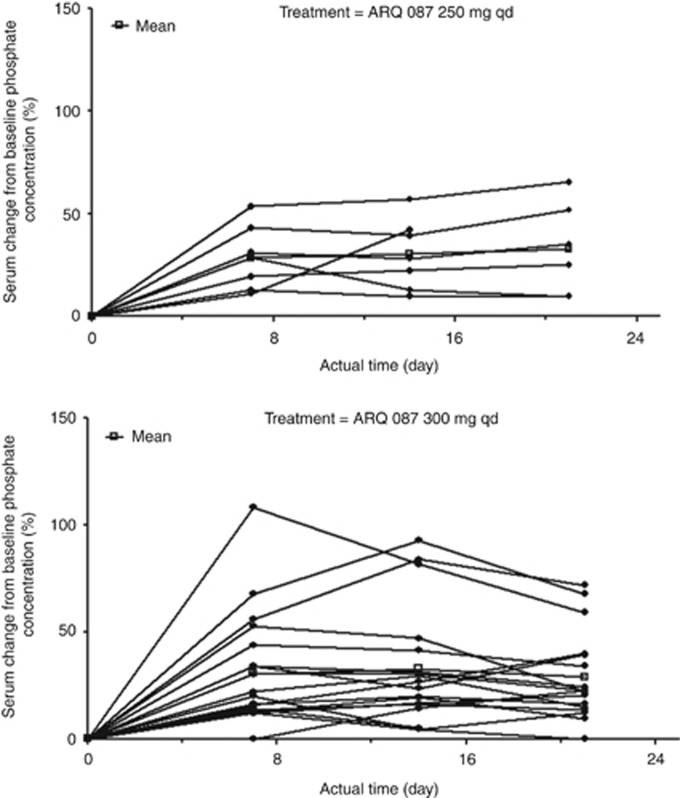
**Serum phosphate per cent change in concentration from baseline in patients doses at 250 mg QD and 300 mg QD.**

Serum phosphate and plasma FGF19, 21, and 23 data were assessed. There was an increase from baseline serum phosphate concentration with increasing ARQ 087 doses. For all dose levels a statistically significant (*P*-value <0.001) exposure response was observed between ARQ 087 exposure (AUC_0–24_) and phosphate PD parameters (*R*_max_, %B *R*_max_; Supplementary Figure 1a). At doses of 250 mg QD and 300 mg QD, the RP2D, phosphate levels tended to increase over the course of Cycle 1 of treatment with ARQ 087 (Figure 2). The time of maximum observed phosphate response (*R*_max_; reflected by *T*_max_) was variable with no apparent dose correlation (median *T*_max_ ranged from 3.0 to 21.0 h).

For the 150–425 mg dose levels, Day 22 plasma ARQ 087 exposure (AUC_0–24_) showed statistically significant (*P*-value=0.028) exposure response with FGF19 *R*_max_ (Supplementary Figure 1b). Higher ARQ 087 exposures were associated with higher increases from baseline in FGF19 levels (Table 3). Similar trends were observed for plasma FGF21 at the 150–425 mg dose levels, where a statistically significant (*P*-value=0.025) exposure-response between ARQ 087 exposure (AUC_0–24_) and FGF21 *R*_max_ was observed (Supplementary Figure 1c). There was no statistically significant positive exposure–response correlation between ARQ 087 and plasma FGF23. Although plasma ARQ 087 concentrations appeared to be saturated at doses above 300 mg, no apparent saturation of effect was detected on serum phosphate or plasma FGF19 responses.

Because of assay optimisation challenges and the limited amount of data analysed, we cannot draw conclusions from the pre- and post-treatment biopsy samples tested for expression of pFGFR, pFRS2, and pERK.

### Antitumour activity

**Figure 3:**
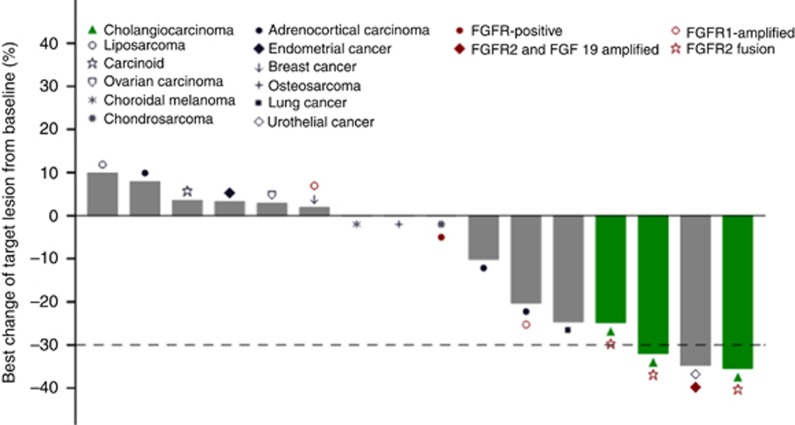
**Relative change from baseline in target lesion size (at best tumour response).** Shown is the per cent change of the lowest sum of the target lesions from baseline for patients who were on treatment for ⩾16 weeks. Seven patients had documented FGFR genetic alterations.

Of 80 patients enrolled, 67 were evaluable for tumour response. Three patients achieved confirmed partial response (PR) and twenty-six had best response of stable disease (SD). Sixteen patients, including seven patients whose tumours had FGFR genetic alterations, received therapy for ⩾16 weeks (Figure 3). No response was observed in two patients with PDGFR/KIT mutations.

Ten patients with response-evaluable iCCA were treated with ARQ 087, seven at 300 mg QD and three at 400 mg QD. In the five iCCA patients with FGFR2 fusions (BICC1 (*n*=2), KIAA1217, TACC1, CCDC6), two confirmed PRs and a SD (25% tumour reduction) of 24–41 weeks duration were observed. In the remaining two patients with FGFR2 fusions and five iCCA patients without FGFR aberrations, progressive disease was the best response.

Two patients with FGFR amplification had clinical benefit with reduction in tumour burden. A patient with urothelial cancer with FGFR2 (copy number (CN)=11) and FGF19 (CN=19) amplification had a confirmed PR (35% tumour reduction) and completed 40 weeks of treatment, and another patient with adrenocortical carcinoma with FGFR1 amplification had SD with a maximum tumour reduction of 20% and was on study for 3.5 years.

Two patients without apparent FGFR genetic alterations bear mentioning. A patient with squamous NSCLC with Src amplification, who had received four prior regimens of systemic therapy, had SD with a 25% tumour reduction and received 36 weeks of ARQ 087. The second patient with adrenocortical carcinoma remains on study treatment for 12 months; her disease has been stable with a maximum tumour reduction of 10%.

## Discussion

ARQ 087 is an orally bioavailable, ATP-competitive, selective FGFR inhibitor with limited activity against other kinases (Hall *et al*, 2016). The toxicity profile of ARQ 087 differs from that expected of other potent FGFR inhibitors.

In this first-in-human study, ARQ 087 was tolerated with manageable toxicities. Reversible increase in AST was the only DLT, which defined the MTD of 400 mg QD. At the RP2D for ARQ 087 of 300 mg QD based on safety and PK data, the most frequent drug-related AEs included nausea (42%), fatigue (32%), ALT increase (26%), and AST increase (21%). In the majority of patients with elevated hepatic transaminases, enzyme levels plateaued despite continued therapy, and only 2 out of 19 (11%) of patients required dose interruption or dose reduction at the RP2D. The observed toxicity profile of ARQ 087 was remarkable for the lack of clinically significant hyperphosphataemia, a toxicity and biomarker of FGFR inhibition typically associated with pan-FGFR inhibitors (Andre *et al*, 2013b; Dienstmann *et al*, 2014; Tabernero *et al*, 2015; Nogova *et al*, 2017). Hyperphosphataemia, investigator defined as grade 1, occurred in only 5% of patients treated with ARQ 087. Dose-dependent increase in serum phosphate was observed in patients, but in the majority of cases levels remained within the upper limit normal range, and no dose interruptions or modifications of ARQ 087 were required at the RP2D.

Hyperphosphataemia associated with FGFR inhibition appears to be mediated by FGF23 signalling through FGFR1 (Shimada *et al*, 2004; Cheng *et al*, 2011). Although the exact mechanism is not known, the modest effect of ARQ 087 on phosphate metabolism may be related to its selectivity towards FGFR2. Biochemically ARQ 087 inhibition appears only slightly more potent towards FGFR2 (IC50 1.8 nM) than FGFR1/3 (IC50 4.5 nM). However, in cancer cell lines as well as in Ba/F3 cell lines engineered to be dependent on FGFR signalling, ARQ 087 shows reduced potency towards FGFR1/3 (Hall *et al*, 2016). We hypothesise that considering the lack of clinically significant hyperphosphataemia, the relative sparing of FGFR1, and the lack of clear dose-related increase in FGF23, ARQ 087 may be in the unique position of potently inhibiting cancers with FGFR2 dysregulation without causing hyperphosphataemia. Significant hyperphosphataemia without a dose-dependent change in FGF23 has been observed with other selective FGFR inhibitors (Tabernero *et al*, 2015). Of relevance is the dose-dependent response evident with FGF19, suggesting that for ARQ 087 FGF19 might potentially have utility as a biomarker of effective FGFR inhibition.

Other frequently reported AEs related to FGFR inhibitors, including ocular, skin, nail, and mucosal toxicity, were observed in patients treated with ARQ 087, although they occurred infrequently and were of low grade; none required dose modification. A number of selective and non-selective FGFR tyrosine kinase inhibitors have been evaluated in various cancers harbouring FGFR genetic alterations (Andre *et al*, 2013a, b; Soria *et al*, 2014; Tabernero *et al*, 2015; Nogova *et al*, 2017; Saka *et al*, 2017). In general, toxicity profiles of selective FGFR inhibitors fare better compared with non-selective multi-kinase inhibitors, with fewer off-target effects attributable to vascular endothelial growth factor receptor and platelet-derived growth factor receptor (PDGFR) inhibition. In the results from the phase I study of the selective FGFR inhibitor JNJ-42756493 (Tabernero *et al*, 2015), the most common AEs were hyperphosphataemia (65%), asthenia (55%), dry mouth (45%), nail toxicity (35%), constipation (34%), and decreased appetite (32%). Dose-dependent hyperphosphataemia required frequent dose interruptions (31%) and modifications (8%). A similar safety profile was seen with the pan-FGFR inhibitor BGJ398, with dose adjustments or interruptions for hyperphosphataemia required in 50% of patients at the RP2D (Nogova *et al*, 2017). In patients with advanced solid tumours treated with the FGFR1,2,3 inhibitor AZD3547, the common treatment-related AEs included fatigue, mucositis, nausea, CSR, and hyperphosphataemia (Andre *et al*, 2013b; Saka *et al*, 2017).

ARQ 087 has a half-life of 5 days, with accumulation following repeat dosing. There were linear PK up to 250 mg QD, but nonlinear thereafter, with apparent saturation in absorption that appeared unaffected by fed conditions. At the RP2D of 300 mg QD, steady-state *C*_min_ was equivalent to or above the concentration where consistent antitumour activity was observed in preclinical models (Hall *et al*, 2016).

Among 16 patients with durable response ⩾16 weeks, seven patients had tumours with FGFR pathway alterations. In these seven patients, three PRs (two with FGFR2 fusion-positive iCCA and one with urothelial cancer with FGFR2 and FGF19 amplification) and two durable SDs with tumour reduction (FGFR2 fusion-positive iCCA and adrenocortical carcinoma with FGFR1 amplification) were observed.

In conclusion, in this Phase 1 study, ARQ 087 was well tolerated with manageable toxicities in a non-selected patient population, and demonstrated single-agent antitumour activity in heavily pretreated patients with specific FGFR genetic alterations. In addition to phosphate, our study suggested that FGF19 might be another biomarker of effective pharmacologic target inhibition. Currently, ARQ 087 is in Phase 2 clinical trial in patients with genetically defined advanced solid tumours such as iCCA, urothelial, and adrenocortical adenocarcinomas. A pivotal study of ARQ 087 will be conducted to further evaluate efficacy of the drug in patients with FGFR2 gene fusion-positive iCCA.

## Figures and Tables

**Table 1:** Patient demographic and baseline characteristics

**Demographics and baseline parameters**	**All patients** ***n*****=80 (%)**
**Age (years)**	
Median (Min, Max)	65 (20, 79)
**Sex**	
Female	47 (59%)
Male	33 (41%)
**Race**	
African American	7 (9%)
Other	4 (5%)
White	69 (86%)
**ECOG**	
0	26 (33%)
1	51 (64%)
2	3 (4%)
**No. of prior systemic regimens**	
Median (Min, Max)	3 (0, 18)
**Cancer type**	
Adrenocortical carcinoma	4 (5%)
Lung cancer	5 (6%)
Colon cancer	7 (9%)
Ovarian carcinoma	7 (9%)
Breast cancer	11 (14%)
Intrahepatic cholangiocarcinoma	12 (15%)
Other	34 (42%)

Abbreviations: ECOG=Eastern Cooperative Oncology Group; Max=maximum; Min=minimum.

**Table 2:** Most common TEAEs (⩾10% of all patients) for escalation and RP2D dose levels

					**All subjects (*****n*****=80; %)**	
**Preferred terms**	**25 mg QOD–200 mg QD (*****N*****=29; %)**	**250 mg QD and 325 mg QD (*****N*****=13; %)**	**400 mg QD–425 mg QD (*****N*****=19)**	**300 mg QD (*****N*****=19; %)**	**Grade** ⩾**3**	**All grades**
Any TEAE	28 (97%)	13 (100%)	19 (100%)	19 (100%)	40 (50%)	79 (99%)
Grade ⩾3 ARQ 087-related AEs	3 (10%)	3 (23%)	6 (32%)	2 (11%)	14 (18%)	70 (88%)
TEAE leading to treatment interruption	10 (34%)	7 (54%)	11 (58%)	3 (16%)	20 (25%)	31 (39%)
TEAE leading to dose reduction	0	2 (15%)	3 (16%)	1 (5%)	1 (5%)	6 (8%)
TEAE leading to treatment discontinuation	1 (3%)	0	5 (26%)	2 (11%)	3 (4%)	8 (10%)
Any SAE	9 (31%)	2 (15%)	6 (32%)	3 (16%)	20 (25%)	20 (25%)
Fatigue	18 (62%)	8 (62%)	11 (58%)	9 (47%)	5 (6%)	46 (58%)
Nausea	14 (48%)	8 (62%)	13 (68%)	8 (42%)	1 (1%)	43 (54%)
Aspartate aminotransferase increased	7 (24%)	7 (54%)	10 (53%)	5 (26%)	12 (15%)	29 (36%)
Diarrhoea	6 (21%)	5 (38%)	9 (47%)	3 (16%)	0	23 (29%)
Decreased appetite	11 (38%)	3 (23%)	6 (32%)	3 (16%)	1 (1%)	23 (029%)
Vomiting	10 (34%)	2 (15%)	7 (37%)	3 (16%)	1 (1%)	22 (28%)
Constipation	5 (17%)	4 (31%)	7 (37%)	4 (21%)	0	20 (25%)
Dry mouth	3 (10%)	1 (8%)	6 (32%)	4 (21%)	0	14 (18%)
Alanine aminotransferase increased	2 (7%)	3 (23%)	4 (21%)	5 (26%)	3 (4%)	14 (18%)
Anaemia	7 (24%)	3 (23%)	0	2 (11%)	2 (3%)	12 (15%)
Blood creatinine increased	4 (14%)	1 (8%)	4 (21%)	2 (11%)	0	11 (14%)
Hypoalbuminaemia	6 (21%)	4 (31%)	1 (5%)	0	1 (1%)	11 (14%)
Blood alkaline phosphatase increased	8 (28%)	2 (15%)	1 (5%)	0	1 (1%)	11 (14%)
Blood lactate dehydrogenase increased	5 (17%)	2 (15%)	3 (16%)	0	1 (1%)	10 (13%)
Dry skin	2 (7%)	2 (15%)	5 (26%)	0	0	9 (11%)
Dysgeusia	3 (10%)	3 (23%)	3 (16%)	0	0	9 (11%)
Anxiety	4 (14%)	3 (23%)	2 (11%)	0	0	9 (11%)
Dizziness	1 (3%)	1 (8%)	5 (26%)	1 (5%)	0	8 (10%)
Dyspepsia	3 (10%)	0	3 (16%)	2 (11%)	0	8 (10%)
Dyspnoea	6 (21%)	0	2 (11%)	0	3 (4%)	8 (10%)

Abbreviations: QD=daily; QOD=every other day; RP2D=recommended phase 2 dose; SAE=serious adverse event; TEAE=treatment-emergent adverse event.

**Table 3:** Pharmacokinetic and pharmacodynamic parameters of ARQ 087 after single and multiple oral doses of ARQ 087

	**ARQ 087 treatment**			
	**100 mg QD**	**200 mg QD**	**300 mg QD**	**400 mg QD**
**Mean (CV%)**	**Plasma ARQ 087**			
**PK Day 1**				
*N*	4	5	11	5
*C*_max_ (ng ml^−1^)	61.65 (34.2)	106.4 (46.3)	164.2 (74.7)	176.4 (24.3)
AUC_0–24_^a^ (ng.h ml^−1^)	1099.72 (36.8)	1820.51 (43.4)	3681.63 (53.2)^b^	2949.84 (31.2)
AUC_last_^c^ (ng.h ml^−1^)	3274.13 (39.7)	1820.51 (43.4)	1940.87 (97.4)	2949.84 (31.2)
*T*_max_^d^ (h)	17.18 (5.97, 46.82)	11.78 (8.00, 23.68)	7.95 (4.00, 24.05)	6.08 (5.93, 22.78)
**PK Day 22**				
*N*	3	5	8	5
*C*_max_ (ng ml^−1^)	330.0 (66.0)	630.0 (12.1)	963.1 (44.6)	913.0 (37.9)
AUC_0–24_^e^ (ng.h ml^−1^)	6689.65 (64.3)	13 584.53 (15.4)^f^	20 339.47 (43.7)	19 887.28 (44.9)
AUC_last_ (ng.h ml^−1^)	6689.65 (64.3)	12 109.14 (31.1)	20 339.47 (43.7)	19 887.28 (44.9)
*C*_min_ (ng ml^−1^)	248.7 (66.8)	506.0 (16.4)	776.3 (43.5)	744.4 (48.1)
*T*_max_^d^ (h)	11.77 (3.92, 23.17)	6.00 (4.00, 9.82)	5.99 (3.83, 22.12)	6.08 (4.00, 22.02)
*T*_min_^d^ (h)	0.92 (0.00, 9.77)	1.00 (0.00, 23.87)	1.06 (0.00, 10.00)	1.00 (0.00, 12.13)
RA *C*_max_	4.93 (32.4)	6.66 (30.5)	9.69 (75.5)	5.24 (32.4)
RA AUC_0–24_	5.45 (31.5)	9.21 (9.3)	8.42 (51.7)	6.9 (41.6)
**Mean (CV%)**	**Serum phosphate and plasma FGF19**			
**PD Cycle 1**				
*N* (serum phosphate/FGF19)	4/4	5/5	19/13	5/5
Phosphate *BR*_max_ (mg dl^−1^)	0.73 (90.0)	0.95 (78.9)	1.25 (53.8)	1.57 (57.2)
FGF19 *BR*_max_ (pg ml^−1^)	186.1 (93.2)	255.4 (127.6)	220.3 (87.7)	371.5 (319.7)

Abbreviations: AUC=area under the plasma concentration–time curve; BR=maximum change from baseline response value; calculated as *R*_max_—*B*, where *R*=maximum response and B=baseline; PD=pharmacodynamics; PK=pharmacokinetics; RA=accumulation ratio; RP2D=recommended phase 2 dose.

Note: on Day 22, the profiles of Subjects 51, 61, 63, and 64 were excluded because of dose reduction after Day 1 or less than 90% compliance to scheduled dosing; 300 mg QD is the RP2D/Expanded cohort.

a For ARQ 087 dose of 150–425 mg, AUC_last_≈AUC_0–24_ since *T*_last_ ranged between 21.9 and 25.3 h on Day 1 (except for Subjects 20 and 21 in the 150 mg cohort).

b *n*=4, AUC_0–24_ not calculated for Subjects 69, 70, 72, 77, 78, 79, and 85 (*T*_last_ ranged from 9.7 to 10.1 h).

c The last blood draw for ARQ 087 25–100 mg was taken 72 h post-dose, whereas the last blood draw was taken 24 h post-dose for ARQ 087 150–425 mg.

d Median (Min, Max).

e AUC_last_≈AUC_0–24_ since *T*_last_ ranged between 22.0 and 29.3 h on Day 22.

f *n*=4, AUC_0–24_ not calculated for Subject 28 (*T*_last_=11.8 h).
