# Supplementary figures and images for: A Phase 1 study of ARQ 087, an oral pan-FGFR inhibitor in patients with advanced solid tumours
Source: Br J Cancer. 2017 Oct 3;117(11):1592–9. doi: 10.1038/bjc.2017.330 (PMC5729432; doi:10.1038/bjc.2017.330)

## **Supplementary** **Figure 1. Serum Phosphate and Plasma FGF PK/PD Correlation Scatterplots**


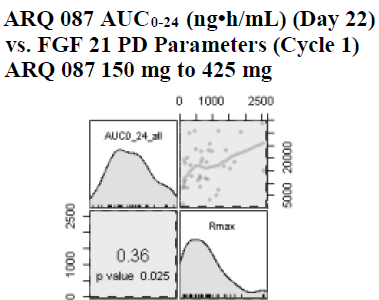

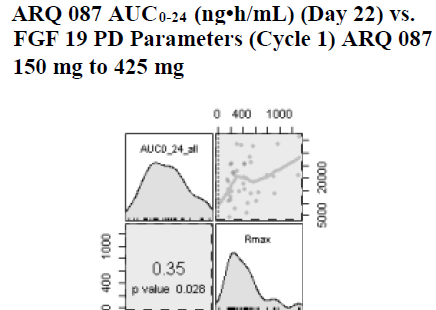

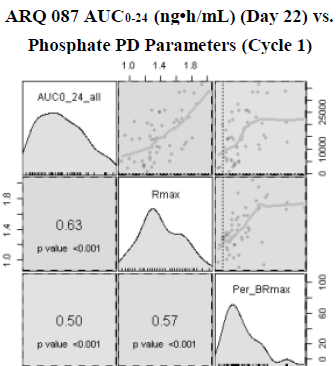


b.

a.

c.

Supplement: Supplementary Figure 1 [file bjc2017330x1.docx]
