# Supplementary material for: A Phase 1 study of ARQ 087, an oral pan-FGFR inhibitor in patients with advanced solid tumours
Source: Br J Cancer. 2017 Oct 3;117(11):1592–9. doi: 10.1038/bjc.2017.330 (PMC5729432; doi:10.1038/bjc.2017.330)
Supplement: Supplementary Table 1 [file bjc2017330x2.docx]

**Supplementary Table 1. Most Common ARQ 87-related Adverse Events (≥ 10% of all patients) for Escalation and RP2D Dose Levels**

| **Adverse Events** | **25 mg QOD- 200 mg QD**  **(N=29) (%)** | **250 mg &**  **325 mg QD**  **(N=13) (%)** | **400 mg &**  **425 mg QD**  **(N=19) (%)** | **300 mg QD**  **(N=19) (%)** | **All Subjects  (N=80)** **(%)**  **Grade ≥3 All grades** | |
| --- | --- | --- | --- | --- | --- | --- |
| **ARQ 087-related AEs** | 23 (79%) | 12 (92%) | 18 (95%) | 17 (89%) | 14 (18%) | 70 (88%) |
| **Grade ≥3 ARQ 087-related AEs** | 3 (10%) | 3 (23%) | 6 (32%) | 2 (11%) | 14 (18%) | 70 (88%) |
| **ARQ 087-related SAE** | 0 | 0 | 1 (5%) | 1 (5 %) | 2 (3%) | 2 (3%) |
| ARQ 087-related AEs leading to treatment interruption | 3 (10%) | 5 (38%) | 9 (47%) | 3 (16%) | 12 (15%) | 20 (25%) |
| ARQ 087-related AEs leading to dose reduction | 0 | 2 (15%) | 3 (16%) | 1 (5%) | 6 (8%) | 6 (8%) |
| ARQ 087-related AEs leading to treatment discontinuation | 0 | 0 | 3 (16%) | 0 | 3 (4%) | 3 (4%) |
| Fatigue | 14 (48%) | 8 (62%) | 11 (58%) | 6 (32%) | 3 (4%) | 39 (49%) |
| Nausea | 11 (38%) | 6 (46%) | 12 (63%) | 8 (42%) | 0 | 37 (46%) |
| Aspartate aminotransferase increased | 4 (14%) | 7 (54%) | 9 (47%) | 4 (21%) | 9 (11%) | 24 (30%) |
| Diarrhea | 3 (10%) | 5 (38%) | 8 (42%) | 2 (11%) | 0 | 18 (23%) |
| Decreased appetite | 5 (17%) | 3 (23%) | 5 (26%) | 2 (11%) | 1 (1%) | 15 (19%) |
| Vomiting | 6 (21%) | 2 (15%) | 5 (26%) | 2 (11%) | 0 | 15 (19%) |
| Alanine aminotransferase increased | 2 (7%) | 3 (23%) | 4 (21%) | 5 (26%) | 3 (4%) | 14 (18%) |
| Constipation | 3 (10%) | 4 (31%) | 5 (26%) | 0 | 0 | 12 (15%) |
| Dry mouth | 1 (3%) | 1 (8%) | 5 (26%) | 2 (11%) | 0 | 9 (11%) |
| Dysgeusia | 3 (10%) | 3 (23%) | 3 (16%) | 0 | 0 | 9 (11%) |
| **ARQ 087-related AEs of special interest** |  |  |  |  |  |  |
| Hyperphosphatemia*** | 1 (3%) | 0 | 1 (5%) | 2 (11%) | 0 | 4 (5%) |
| Eye toxicity* | 0 | 1 (8%) | 2 (11%) | 0 | 0 | 3 (4%) |
| Nail toxicity** | 1 (3%) | 1 (8%) | 1 (5%) | 0 | 0 | 3 (4%) |
| Abnormal LFTs | 4 (14%) | 7 (54%) | 9 (47%) | 5 (26%) | 9 (11%) | 25 (31%) |
| Cardiac toxicity **** | 0 | 0 | 2 (11%) | 1 (5%) | 2 (3%) | 3 (4%) |
| Abbreviations: TEAEs – treatment-emergent adverse events; AE – adverse event; SAE – serious adverse event;  Percentages were calculated using the number of patients in the safety population of each group as denominator. Recurring events are counted only once for each patient.  TEAEs include any adverse event that occurred from the time of the administration of the first dose of ARQ 087 to within 30 days after the last dose administration.  No ARQ 087-related deaths were reported.  *Eye toxicity includes 1 patient each with dry eye, blurred vision and visual impairment. No patients had grade ≥3 eye toxicity.  **Nail toxicity includes 1 patient each with nail avulsion, nail discoloration, and onychomycosis.  ***Hyperphosphatemia is reported per the Investigator assessment grade.  ****Cardiac toxicity includes ECG prolonged QTc, and non-ischemic diffuse T-wave inversion. | | | | | | |
