# Supplementary material for: A Phase 1 study of ARQ 087, an oral pan-FGFR inhibitor in patients with advanced solid tumours
Source: Br J Cancer. 2017 Oct 3;117(11):1592–9. doi: 10.1038/bjc.2017.330 (PMC5729432; doi:10.1038/bjc.2017.330)
Supplement: Supplementary Table 2 [file bjc2017330x3.docx]

Supplementary Table 2. Mean (CV%) Plasma Pharmacokinetic Parameters of ARQ 087 After Single (Day 1) and Multiple (Day 22) Oral Doses of ARQ 087

| Mean (CV%) | Plasma ARQ 087 | | | | | | | | | | | |
| --- | --- | --- | --- | --- | --- | --- | --- | --- | --- | --- | --- | --- |
|  | ARQ 087 Treatment | | | | | | | | | | | |
|  | 25 mg QOD | 25 mg QD | 50 mg QD | 100 mg QD | 150 mg QD | 200 mg QD | 250 mg QD | 300 mg QD | 325 mg QD | 400 mg QD | 400 mg QD Fed | 425 mg QD |
| Day 1 | | | | | | | | | | | | |
| N | 3 | 6 | 6 | 4 | 5 | 5 | 7 | 11 | 6 | 5 | 7 | 7 |
| C_max_  (ng/mL) | 5.950 (88.6) | 15.97 (35.9) | 36.02 (33.6) | 61.65 (34.2) | 97.24 (44.4) | 106.4 (46.3) | 154.3 (15.8) | 164.2 (74.7) | 204.3 (37.3) | 176.4 (24.3) | 238.6 (32.8) | 184.4 (44.3) |
| AUC_0-24_^a^  (ng•h/mL) | 84.59 (140.4) | 297.75 (42.4) | 656.39 (37.0) | 1099.72 (36.8) | 1795.28 (49.0) | 1820.51 (43.4) | 2680.68 (19.9) | 3681.63 (53.2)^f^ | 3430.90 (32.8) | 2949.84 (31.2) | 3872.43 (33.5) | 3244.95 (48.3) |
| AUC_last_^b^  (ng•h/mL) | 303.20 (108.0) | 900.75 (34.9) | 2019.97 (29.6) | 3274.13 (39.7) | 2843.11 (81.8) | 1820.51 (43.4) | 2680.68 (19.9) | 1940.87 (97.4) | 3430.90 (32.8) | 2949.84 (31.2) | 3872.43 (33.5) | 3244.95 (48.3) |
| T_max_^c^  (h) | 47.00 (8.10, 48.03) | 17.46 (8.07, 24.23) | 11.75 (6.12, 25.00) | 17.18 (5.97, 46.82) | 11.78 (7.87, 23.95) | 11.78  (8.00, 23.68) | 22.97 (6.00, 24.00) | 7.95 (4.00, 24.05) | 10.77 (4.00, 24.28) | 6.08  (5.93, 22.78) | 11.75  (7.82, 23.35) | 7.92  (3.97, 23.15) |
| Day 22 | | | | | | | | | | | | |
| N | 3 | 4 | 5 | 3 | 5 | 5 | 5 | 8 | 5 | 5 | 4 | 4 |
| C_max_  (ng/mL) | 93.43 (54.6) | 171.0 (20.4) | 308.4 (26.0) | 330.0 (66.0) | 613.6 (41.7) | 630.0 (12.1) | 1031 (31.8) | 963.1 (44.6) | 769.6 (48.4) | 913.0 (37.9) | 1086 (42.9) | 1108 (28.1) |
| AUC_0-24_^d^  (ng•h/mL) | 1660.76 (54.9) | 3829.02 (29.8) | 6645.53 (26.4) | 6689.65 (64.3) | 13270.79 (46.2) | 13584.53 (15.4)^e^ | 20584.39 (23.5) | 20339.47  (43.7) | 16041.20 (50.5) | 19887.28 (44.9) | 24265.94 (49.9) | 25396.83 (20.9) |
| AUC_last_  (ng•h/mL) | 1660.76 (54.9) | 3829.02 (29.8) | 6645.53 (26.4) | 6689.65 (64.3) | 13270.79 (46.2) | 12109.14 (31.1) | 20584.39 (23.5) | 20339.47 (43.7) | 16041.20 (50.5) | 19887.28 (44.9) | 24265.94 (49.9) | 25396.83 (20.9) |
| C_min_  (ng/mL) | 54.67 (52.3) | 140.7 (29.8) | 256.6 (29.4) | 248.7 (66.8) | 482.4 (41.0) | 506.0 (16.4) | 786.2 (24.8) | 776.3 (43.5) | 544.4 (53.2) | 744.4 (48.1) | 857.5 (46.2) | 836.5 (32.1) |
| T_max_^c^  (h) | 22.32  (3.97, 24.00) | 5.03  (1.88, 22.08) | 6.08  (1.97, 22.05) | 11.77 (3.92, 23.17) | 5.98  (4.00, 24.00) | 6.00  (4.00,  9.82) | 8.00  (4.00, 23.02) | 5.99  (3.83, 22.12) | 6.00  (4.00, 10.00) | 6.08  (4.00, 22.02) | 5.05  (3.97, 9.93) | 10.88 (5.83, 23.78) |
| T_min_^c^  (h) | 0.00  (0.00, 11.80) | 1.48  (0.00, 3.98) | 1.02  (0.00, 8.00) | 0.92  (0.00, 9.77) | 1.93  (0.00, 11.75) | 1.00  (0.00, 23.87) | 1.00  (0.00, 9.88) | 1.06  (0.00, 10.00) | 1.00  (0.00, 23.08) | 1.00  (0.00, 12.13) | 1.56  (0.00, 8.08) | 1.97  (0.92, 9.95) |
| ^a^ For ARQ 087 dose of 150 mg to 425 mg, AUC_last_ ≈ AUC_0-24_ since T_last_ ranged between 21.9 to 25.3 h on Day 1 (except for Subject 20 and 21 in the 150 mg Cohort).  ^b^ The last blood draw for ARQ 087 25 mg to 100 mg was taken 72 h post-dose, whereas the last blood draw was taken 24 h post-dose for ARQ 087 150 to 425 mg.  ^c^ Median (Min, Max); ^d^ AUC_last_ ≈ AUC_0-24_ since T_last_ ranged between 22.0 to 29.3 h on Day 22.  ^e^ n=4, AUC_0-24_ not calculated for Subject 28 (T_last_=11.8 h).  ^f^ n = 4, AUC_0-24_ not calculated for Subjects 69, 70, 72, 77, 78, 79 and 85 (T_last_ ranged from 9.7 to 10.1 h)  On Day 22, the profiles of Subject 51, 61, 63, 64 were excluded due to dose reduction after Day 1 or less than 90% compliance to scheduled dosing.  300 mg qd = Expanded Cohort | | | | | | | | | | | | |
